# Exposure Optimization Trial for Patients With Medical Implants During MRI Exposure: Balance Between the Completeness and Efficiency

**DOI:** 10.3389/fpubh.2021.793418

**Published:** 2021-12-13

**Authors:** Aiping Yao, Pengfei Yang, Mingjuan Ma, Yunfeng Pei

**Affiliations:** ^1^Department of Information Science and Engineering, Lanzhou University, Lanzhou, China; ^2^Centre for Medical Device Evaluation, National Medical Products Administration, Beijing, China

**Keywords:** RF, medical implant, exposure optimization, MRI, *in silico*

## Abstract

Elongated conductors, such as pacemaker leads, can couple to the MRI radio-frequency (RF) field during MRI scan and cause dangerous tissue heating. By selecting proper RF exposure conditions, the RF-induced power deposition can be suppressed. As the RF-induced power deposition is a complex function of multiple clinical factors, the problem remains how to perform the exposure selection in a comprehensive and efficient way. The purpose of this work is to demonstrate an exposure optimization trail that allows a comprehensive optimization in an efficient and traceable manner. The proposed workflow is demonstrated with a generic 40 cm long cardio pacemaker, major components of the clinical factors are decoupled from the redundant data set using principle component analysis, the optimized exposure condition can not only reduce the *in vivo* power deposition but also maintain good image quality.

## Introduction

Patients with implantable medical devices are usually excluded from the MRI examinations due to the very complicated electromagnetic (EM) environment patients are exposed to during MRI, including static, gradient, and radiofrequency (RF) magnetic fields. The RF magnetic field with frequencies of 64 MHz (1.5 T MRI) and 128 MHz (3.0 T MRI) will induce a strong electric-field in patients based on Faraday law (1, 2). The conductive implants inside the patients will act like an antenna, couple with these induced fields, and deposit the power near the implant electrodes, leading to high local tissue temperature increase (3–5).

Many efforts are done to solve this RF safety problem by modifying the material composition and EM properties of the implanted devices to render them inherently safe for MRI (6, 7), but in most cases, this is not enough. Instead of modifying implanted devices for the MR environment, many explorations are focused on making the MR environment itself safer for existing devices by manipulating the MR exposure conditions (8, 9). On the other hand, the exposure condition selected to reduce the RF-induced heating may at the same time decrease the MRI imaging quality dramatically (10). Therefore, it is important that the exposure condition are optimized so that the RF-induced heating are reduced and at the same time certain MRI imaging quality is reserved.

The RF-induced heating is directly determined by the induced *in vivo* tangential electrical field along with the implant routing (*E*_*tan*_), while the MRI imaging quality can be indicated by the magnetic field strength and homogeneity. For patients with medical implants, the induced *in vivo* electrical field *E*_*tan*_ amounts to a multitude of variables specific to the MRI system (11, 12) (e.g., RF-coil design and manufacturing details), patient anatomy (13, 14), and imaging positions. Therefore, clinical trials performed with a limited number of scenarios are likely to be insufficient to ensure patient safety. It is essential that the exposure optimization be performed in as many relevant clinical scenarios as possible.

In this work, we established an *in silico* exposure optimization trial that comprises a data library with principle component analysis (PCA) to balance between the efficiency and completeness during the exposure optimization procedure. The proposed work-flow is applied to a generic 40-cm long cardio pacemaker under 1.5T MRI RF exposure. Big data containing more than 0.3 billion unique clinical scenarios are selected from the data library. The correlation coefficients between different clinical scenarios are analyzed based on PCA to decouple the major components of the clinical factors which produce significant and unique variation in the implant power deposition. The decoupled major clinical scenarios greatly reduce the data redundantly, therefore, enable a comprehensive and efficient exposure optimization resulting in both good imaging quality and patient safety.

## Materials and Methods

**Figure 1:**
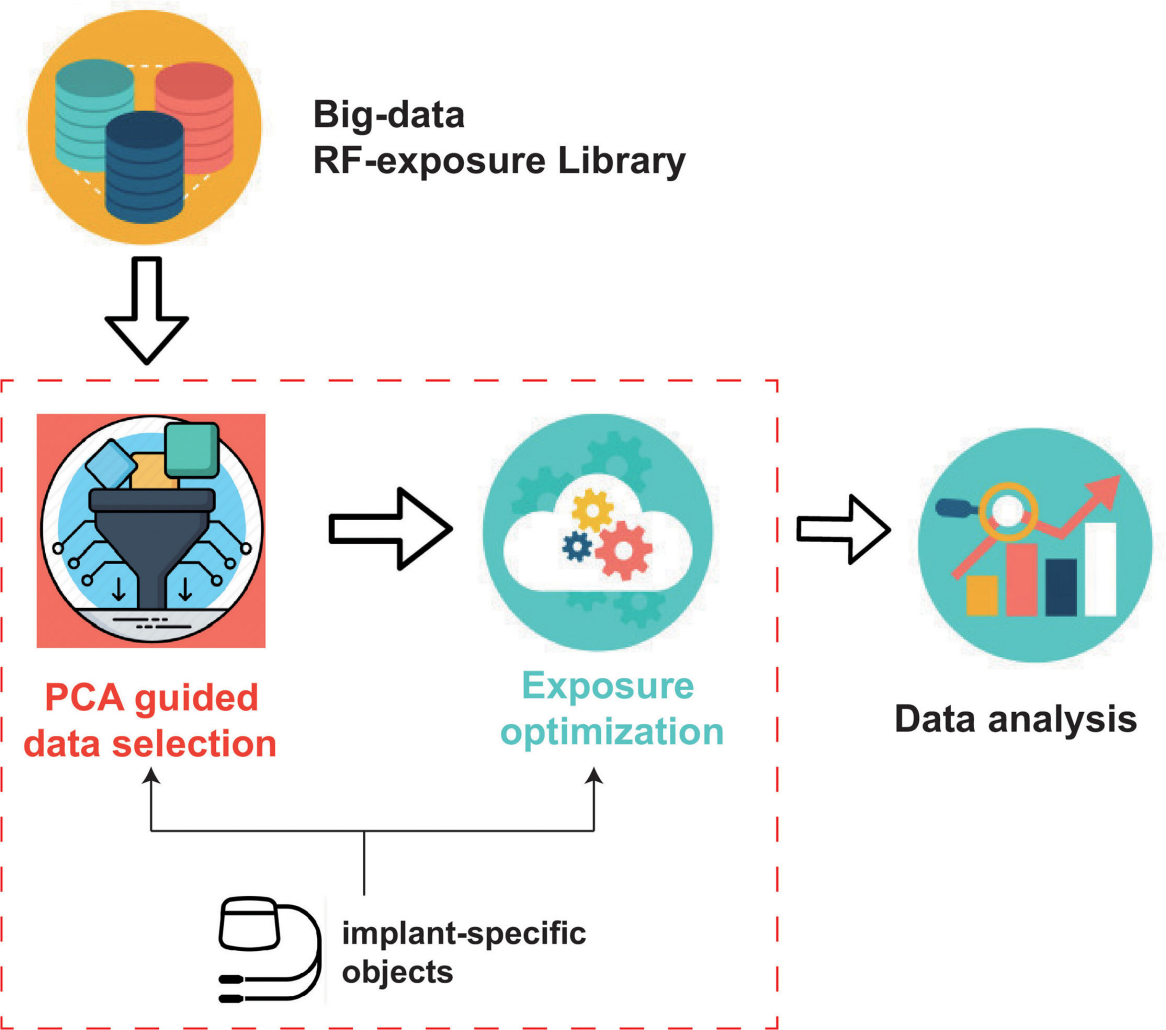
Proposed exposure optimization work-flow.

The proposed framework is illustrated in Figure 1, which is comprised of the following components:

- RF-exposure big-data library: This component provides pre-computed RF-induced field distributions inside a variety of patients during MRI exposure under different clinical scenarios.
- Implant-specific objects: This component includes the digital representations of clinical routings of the implant under test (IUT) and the RF model of the IUT.
- PCA guided data selection: This component uses PCA to get the decoupled clinical factors for an efficient data selection from the data library.
- Exposure Optimization: This component implements exposure optimization to achieve both good imaging quality and patient safety.

### RF-Exposure Big-Data Library

**Figure 2:**
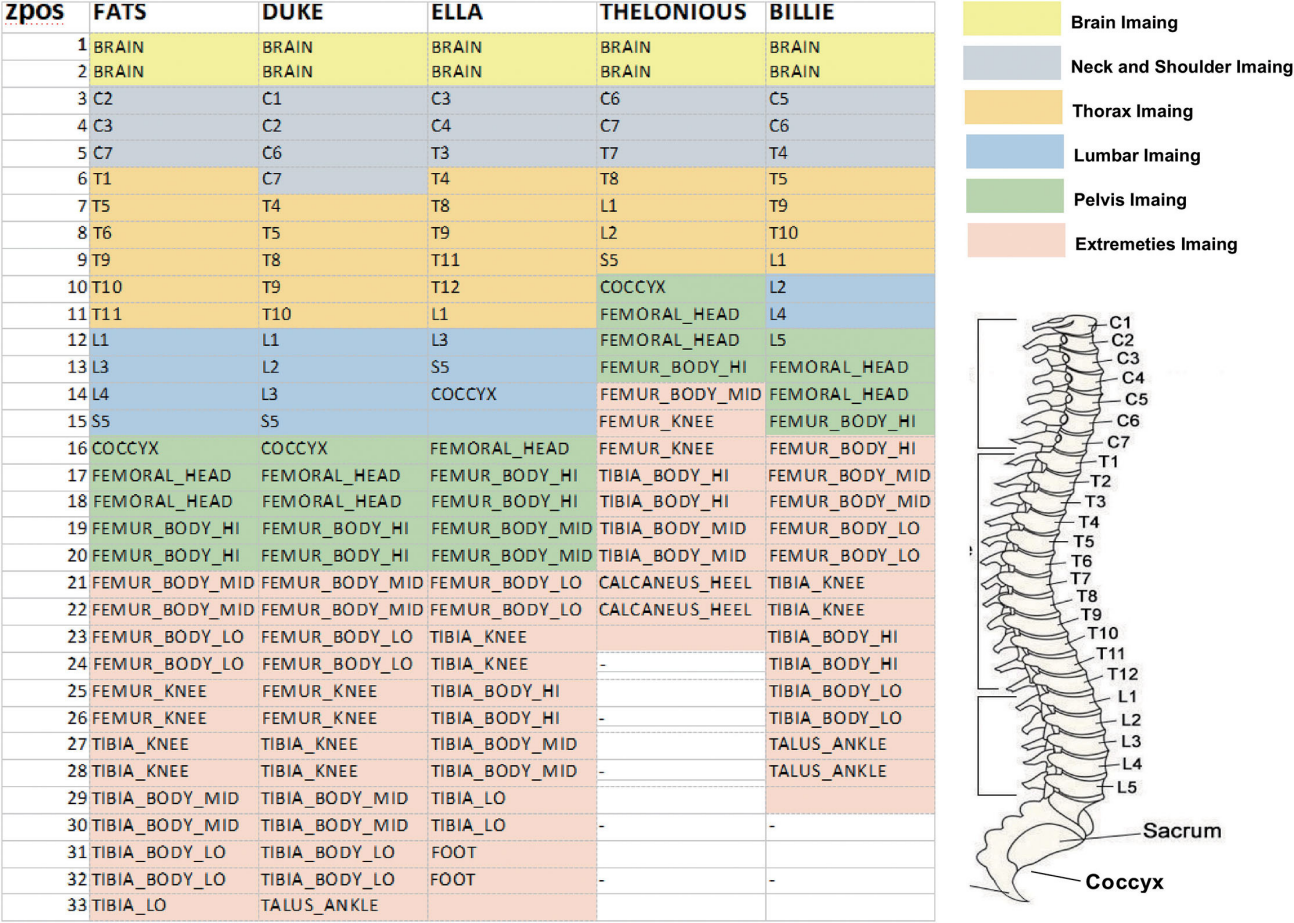
Illustration of the corresponding anatomical marks in each anatomical model for each imaging position (ZPOS), different color represents different imaging range.

Five high-resolution anatomical models from Virtual Population (ViP) (15) representing a large population range are selected and listed in Table 1. 10 RF coils with different coil diameter, and lengths covering the envelope of commercial MRI system are used as the incident RF field source. The geometry of the 10 selected RF coils is listed in Table 2. Each two-channel coil was tuned to resonate at 64 MHz, with selected polarization sweeps included:ϵ ∈ [−45^*o*^, 45^*o*^] with a step of 5^*o*^; τ ∈ [0^*o*^, 180^*o*^] with a step of 10^*o*^, covering a wide range of shimming used in MRI systems. τ and ϵ are two parameters defined to characterize the ellipticity and tilt angle of the field polarization (1). Each anatomical model was placed in the RF coils with imaging positions from head-to-foot with a step size of 10 cm along the longitudinal axis (as FATS is too big for coil No. 1–3, only coil from No. 4–10 are used for FATS). Figure 2 summarized the anatomical marks corresponding to each imaging position (ZPOS) for the five anatomical models.

**Table 1:** Physiological parameters of the five selected anatomical models, obtained from discretized models with a uniform grid size of 0.5 × 0.5 × 0.5*mm*^3^.

**Anatomical model**	**Gender**	**Age**	**Height**	**Weight**	**BMI**
		**(Year)**	**(m)**	**(kg)**	**(Kg/m^**2**^)**
Fats	Male	37	1.82	119	36
Duke	Male	34	1.77	70.2	22.4
Ella	Female	26	1.63	57.3	21.6
Billie	Female	11	1.49	34	15.3
Thelonious	Male	6	1.16	18.6	13.8

**Table 2:** Geometry of the radio frequency (RF) birdcage coil considered in the study.

**Coil no**.	**Diameter**	**Length**	**Number of rungs**	**Shield diameter**	**Frequency**
	**(cm)**	**(cm)**		**(cm)**	**(MHz)**
1	65	50	16	70	64
2	65	60	16	70	64
3	65	70	16	70	64
4	75	40	16	70	64
5	75	50	16	70	64
6	75	60	16	70	64
7	75	70	16	70	64
8	80	50	16	70	64
9	80	60	16	70	64
10	80	70	16	70	64

Computational EM (CEM) simulations were conducted by means of the finite different time domain (FDTD) simulation platform, Sim4Life V6.0 (ZMT Zurich MedTech, Zurich, Switzerland). It was ensured that a steady-state was attained before the simulations were determined. The anatomical models were discretized with a maximum grid size of 2.0 x 2.0 x 2.0 mm^3^, and dielectric tissue properties at 64 MHz (16) were assigned to the tissues.

### Implant-Specific Objects

Three clinical routing groups of the IUT were defined: (i) left and right deep brain stimulator (DBS) routing groups (DBS_*L*_ and DBS_*R*_: the routings run underneath the skin from the proximal ends of the left and right pectoral muscles, along the side of the neck behind the left and right ears, up to the crown of the head, and through the skull, terminating in the distal end of the thalamus); (ii) left and right pacemaker (PM) routing groups (PM_*L*_ and PM_*R*_): the routings run underneath the skin from the proximal end of the left and right pectoral and along the veins, terminating in the distal end of the right heart ventricle; and (iii) left and right spinal cord stimulator (SCS) routing groups (SCS_*L*_ and SCS_*R*_: the routings run underneath the skin from left and right buttocks below the waistline, along with the epidural space from the T10 vertebra, and terminating at the C1 vertebra.

**Algorithm 1:** An algorithm with caption.

Require: M*M is the dimension of the pre-defined power deposition matrix C;
*i* ← 1
*j* ← 1
while *i* ≤ *M* **do**
while *j* ≤ *M* **do**
if *C*_*ij*_ > 0.95 **then**
compress elements i and j in the same compressed group G
else
assign element j to a new compressed group G+1
end **if**
*j* ← *j* + 1
end **while**
*i* ← *i* + 1
end **while**

**Figure 3:**
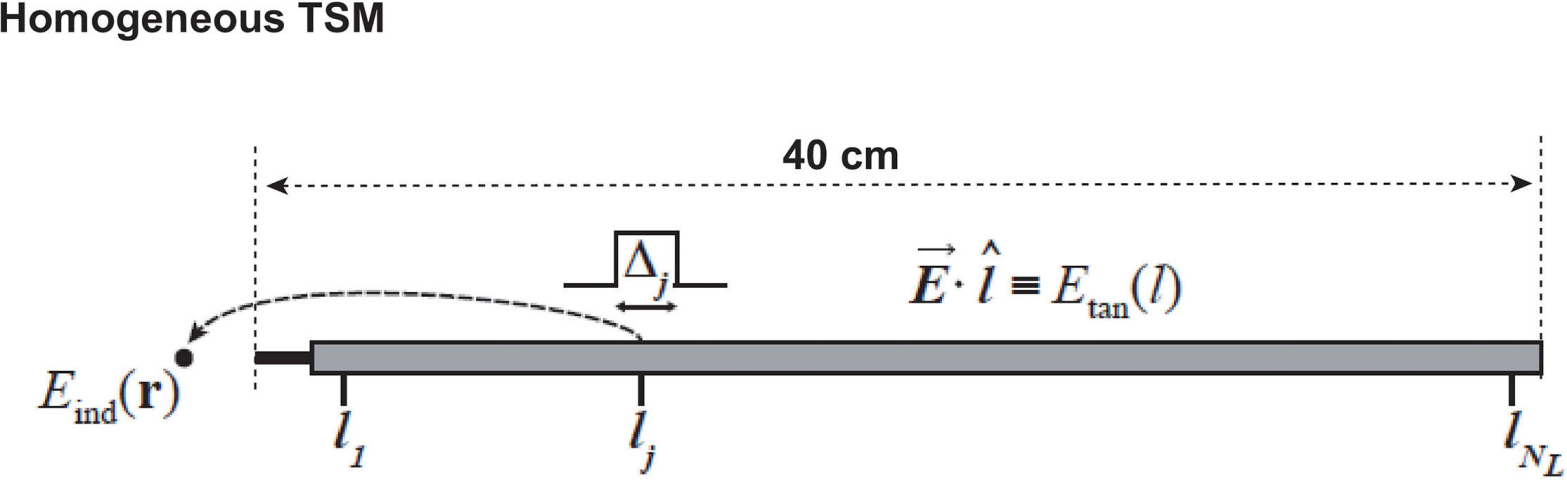
Schematic of the transfer function derived method. *l* is the unit tangential vector along the implant at length *l*.

The RF-model of the IUT defined by the transfer function of the implant can be derived from the technique proposed (17) where the transfer function, henceforth referred to as *h*(*l*), is defined as the locally induced electric field around an electrode with excitation along length *l* of the implant. Figure 3 depicts a schematic of the method, where the generic 40 cm long implant is embedded in a homogeneous tissue simulating medium (TSM) with dielectric properties of σ = 0.47 S/m and ϵ = 78. The tangential component of the local incident electric field, *E*_*tan*_, is coupled with the implant at length *l* and the induced electric field around the implanted electrode at **r**, *E*_*ind*_(***r***), is evaluated as the transfer function *h*(*l*).

### PCA Guided Data Selection

To provide guidance for an efficient RF field data selection, PCA (18) is applied to an implant routing groups, RF coils, and image positions, respectively, to decouple the critical clinical factors from the redundant data. The PCA algorithm performed in this work is defined as follows:

For clinical factors that has M variables (e.g., there are 6 implant routing groups, M = 6), let $A_{i}\in R^{1\times N}$ and $A_{j}\in R^{1\times N}$ be the observed power deposition (*P*_*dep*_) data set for variable *i* and *j*, respectively, the covariance matrix **C**∈*R*^*M*×*M*^ can be obtained through Equation 1:


(1) $$\begin{array}{l} C_{i,j}=\frac{1}{N-1}\left(\frac{A_{i}-hu_{i}^{T}}{\sigma_{i}}\right){\left(\frac{A_{j}-hu_{j}^{T}}{\sigma_{j}}\right)}^{H}\left(i,j\in \left[1,M\right]\right) \end{array}$$

where $u_{i}^{T}$ and σ_*i*_ are the mean and SD of data set $A_{i}\in R^{1\times N}$, $u_{j}^{T}$ and σ_*j*_ are the mean and SD of data set $A_{j}\in R^{1\times N}$. *h* is unity column vector. *H* donate the Hermitian transpose of matrix. Each element on the principal diagonal of the matrix is the correlation of a random variable with itself, which always equals 1.

The RF-induced power deposition *P*_*dep*_ of the implant under each clinical scenario can be estimated from:


(2) $$\begin{array}{l} P_{dep}=\left(\overset{N_{L}}{\underset{j=1}{\sum }}h\left(l_{j}\right)E_{tan}\left(l_{j}\right)\Delta_{j}\right){\left(\overset{N_{L}}{\underset{j=1}{\sum }}h\left(l_{j}\right)E_{tan}\left(l_{j}\right)\Delta_{j}\right)}^{*} \end{array}$$

where *h*_*l*_ is the transfer function of the implant, and *E*_*tan*_(*l*) is the *in vivo* tangential electrical field along the implant trajectory under the selected clinical scenario.

### Exposure Optimization

The RF coil exposure condition can be characterized with poincare shpere parameters ϵ and τ (1). Therefore, different exposure conditions have different ϵ and τ values. When the RF coil is operating under *N* different exposure conditions, these exposure conditions can be represented by poincare sphere parameter vector (ϵ, τ) = [(ϵ^(1)^, τ^1^), (ϵ^(2)^, τ^2^), …, (ϵ^(*N*)^, τ^*N*^)], where (ϵ^(*n*)^, τ^(*n*)^) (*n* ∈ [1, *N*])) is the poincare sphere parameter of the n^*th*^ exposure condition.

For a RF birdcage coil or transmit coil with 2 channels, the total $B_{1}^{+}$ field at each region of interest (ROI) iso-plane can be expressed as the weighted superposition of the $B_{1}^{+}$ field generated by each channel in the RF coil. Let 2 x 1 vector, **b**_**1**_=${\left[b_{1}^{\left(1\right)}\left(\text{r}\right),b_{1}^{\left(2\right)}\left(\text{r}\right)\right]}^{T}$ be the complex $B_{1}^{+}$ field vector, where $b_{1}^{\left(1\right)}\left(\text{r}\right)$ and $b_{1}^{\left(2\right)}\left(\text{r}\right)$ are the complex $B_{1}^{+}$ field generated by the 1*st* and 2*nd* channel of the RF coil at location **r**. Let 2 x 1 vector ${\text{v}}_{\left(\epsilon^{\left(n\right)},\tau^{\left(n\right)}\right)}={\left[v^{\left(1\right)},v^{\left(2\right)}\right]}^{T}$ be the complex excitation vector under exposure condition n, where *v*^(1)^ and *v*^(2)^ are the corresponding complex amplitude of the 1st and 2nd channels. The total $B_{1}^{+}$ field for each specific exposure condition n can then be expressed as follows:


(3) $$\begin{array}{l} \left\|B_{1,\left(\epsilon^{\left(n\right)},\tau^{\left(n\right)}\right)}^{+}\left(r\right)\|=\|b_{1}^{T}v_{\left(\epsilon^{\left(n\right)},\tau^{\left(n\right)}\right)}\right\| \end{array}$$

The coefficient of variation of $\left\|B_{1}^{+}\right\|$, defined as the SD over the mean value, is a commonly accepted figure of merit as a measure of the homogeneity of $\left\|B_{1}^{+}\right\|$, can be obtained through Equation 4:


(4) $$\begin{array}{l} B_{1,cov,\left(\epsilon^{\left(n\right)},\tau^{\left(n\right)}\right)}^{+}=\frac{\sigma }{\left\|\bar{B_{1,\left(\epsilon^{\left(n\right)},\tau^{\left(n\right)}\right)}^{+}}\right\|} \end{array}$$

where σ is the standard deviation of $\left\|B_{1,\left(\epsilon^{\left(n\right)},\tau^{\left(n\right)}\right)}^{+}\right\|$ over the ROI iso-plane. Similar to the *B*_1_ field, tangential electrical field under the *n*_*th*_ exposure condition ${\text{E}}_{\text{tan},\left(\epsilon^{\left(n\right)},\tau \left(n\right)\right)}\left(\text{l}\right)$ can be expressed as the weighted superposition as follows:


(5) $$\begin{array}{l} E_{tan,\left(\epsilon^{\left(n\right)},\tau^{\left(n\right)}\right)}\left(l\right)=e_{tan}v_{\left(\epsilon^{\left(n\right)},\tau^{\left(n\right)}\right)}^{T} \end{array}$$

where ***e***_***tan***_=${\left[e_{tan,1},e_{tan,2}\right]}^{T}$ is the tangential electric field generated by the two RF coil channels. Therefore, the local power deposition at the electrode-tissue interface under this exposure condition can be concisely expressed as follows:


(6) $$\begin{array}{l} P_{dep,\left(\epsilon^{\left(n\right)},\tau^{\left(n\right)}\right)}=W_{0}\|h^{T}E_{tan,\left(\epsilon \left(n\right),\tau \left(n\right)\right)}\left(l\right)\| \end{array}$$

In this work, we selected one specific clinical scenario (anatomical model Duke inside RF coil 6 at the thorax imaging position) to perform the exposure optimization, the following Magnitude Least Squares (MLS) optimization strategy is performed to determine the optimized excitation parameter (ϵ^(*j*)^, τ^(*j*)^):


(7) $$\begin{array}{l} \underset{\left(\epsilon^{\left(j\right)},\tau^{\left(j\right)}\right)}{\min}\|{P_{dep,\left(\epsilon ,\tau \right)}+B_{1,cov\left(\epsilon ,\tau \right)}^{+}-\|B_{1,\left(\epsilon ,\tau \right)}^{+}\|\|}^{2} \end{array}$$

## Results

**Figure 4:**
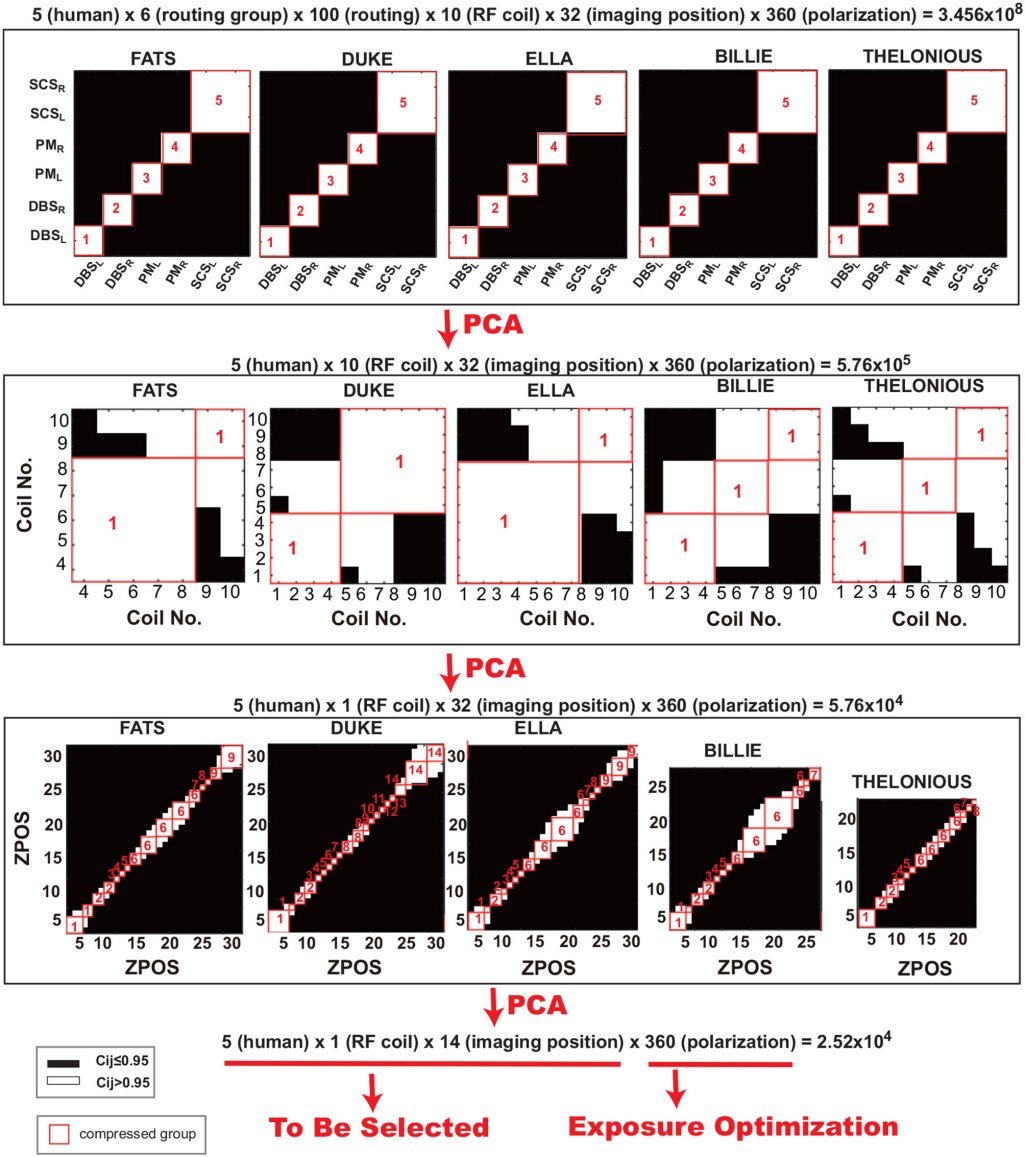
Illustration of the principle component analysis (PCA) guided data compression procedure: covariance matrix *C* of implant routing **(Top Row)**, RF coil **(Middle Row)**, and imaging position **(Botoom Row)** are shown following the data compression order.

Figure 4 demonstrates the PCA-guided clinical factor decoupling procedure. More than 0.3 billion clinical scenarios are contained in the data library, including 5 human models× 6 routing groups × 100 routings for each group × 10 RF coils × 32 imaging positions × 360 exposure polarization. First, to decouple the target implant routing (left-side cardio pacemaker, namely PM_**L**_) from other routings, PCA procedure is performed on the six routing groups. The covariance matrix for the six routing groups (DBS_**L**_, DBS_**R**_, PM_**L**_, PM_**R**_, SCS_**L**_, and SCS_**R**_) are shown on the top row of Figure 4. We define two variables as correlated when *C*_*i,j*_ ≥ 0.95 and shown as white, otherwise, it is considered to be uncorrelated and shown as black. The results show that, for the five anatomical models, all implant routings are independent to each other except for the SCS_**L**_ and SCS_**R**_, this is due to the fact that the IUT is too short (40 cm) to see the separation between left side and right side. Same PCA procedure is performed on the 100 routings in each group. The resulting covariance matrix has all the elements *C*_*i,j*_ bigger than 0.95, therefore, for each routing group, only 1 routing needs to be selected.

In the next setp, the covariance matrix of the RF coils are calculated as shown in the middle row of Figure 4, following the same PCA procedure, the RF coil is compressed to only one, here, we choose RF coil 6. After the selection of the RF coil, the imaging positions are decoupled using the same PCA procedure, as shown in the bottom row of Figure 4. From the covariance matirx we can see that the imaging positions can be compressed to at most 14 groups (e.g., for anatomical model ELLA, imaging positions 0–10 (head to thorax imaging positions) can be compressed as group 1, imaging position 15–20 (pelvis imaging position) may be compressed as group 6, and position 25–30 (extremeties imaging positions) can be compressed as group 9). After the PCA guided data compression procedure, only 0.25 million clinical scenarios are selected from the original more than 0.3 billion data set. Among these selected data sets, the exposure optimization only need to be done among the 70 specific clinical scenarios (5 human × 1 RF coil × 14 imaging positions), as the exposure dimension (360 exposure polarizations) will be compressed by the exposure optimization procedure, where the optimized exposure condition will be selected to maintain patient safety and imaging quality.

**Figure 5:**
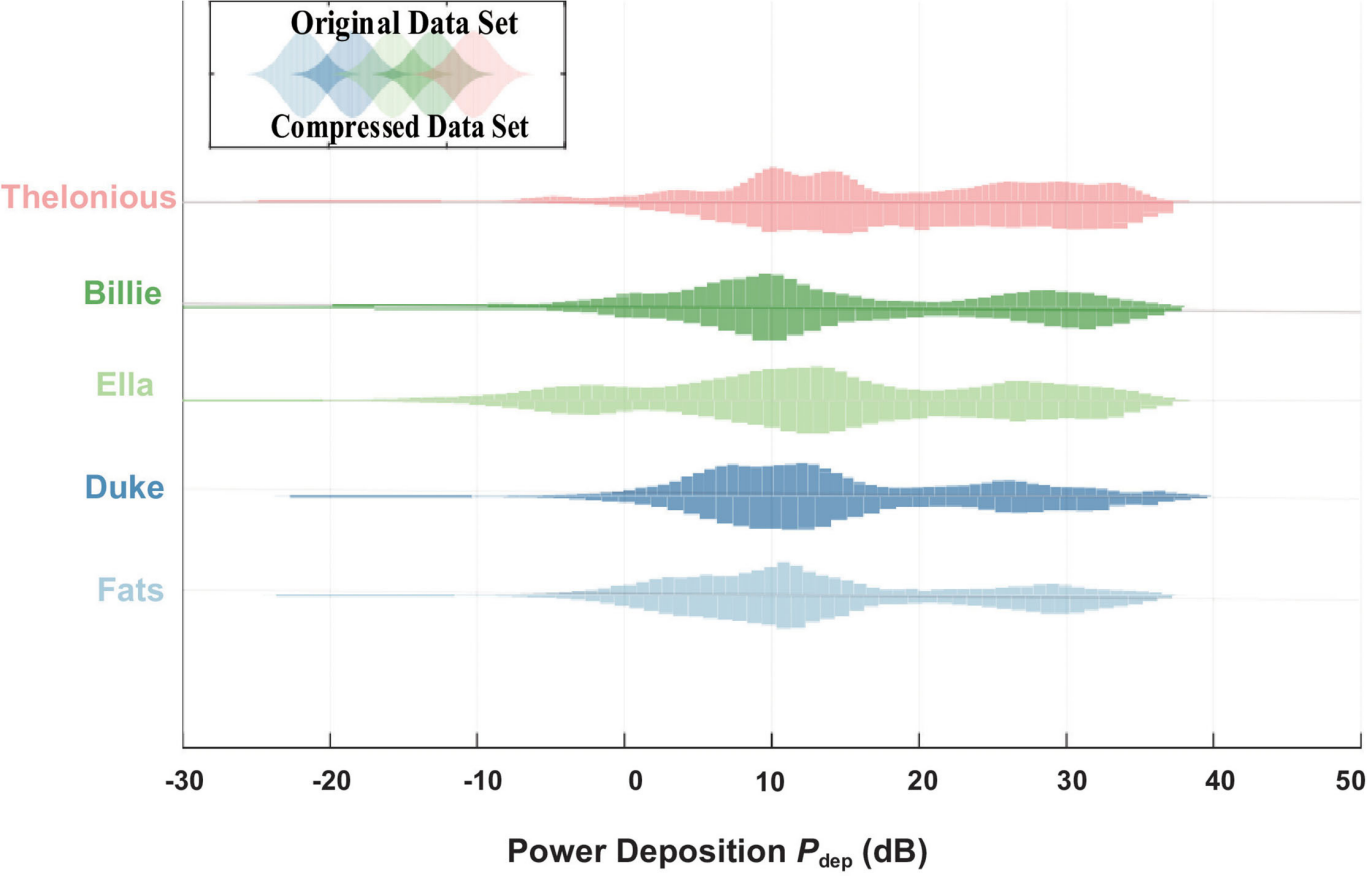
Power deposition (*P*_*dep*_) distribution of the original data set from the data library (up) and compressed data set by PCA (down).

The *in vivo* RF-induced heating of the generic 40 cm implant was estimated with both original clinical scenarios and the selected ones based on PCA guidance. As shown in Figure 5, the power deposition dynamic range of the selected clinical scenarios are the same as those with original clinical scenarios.

**Figure 6:**
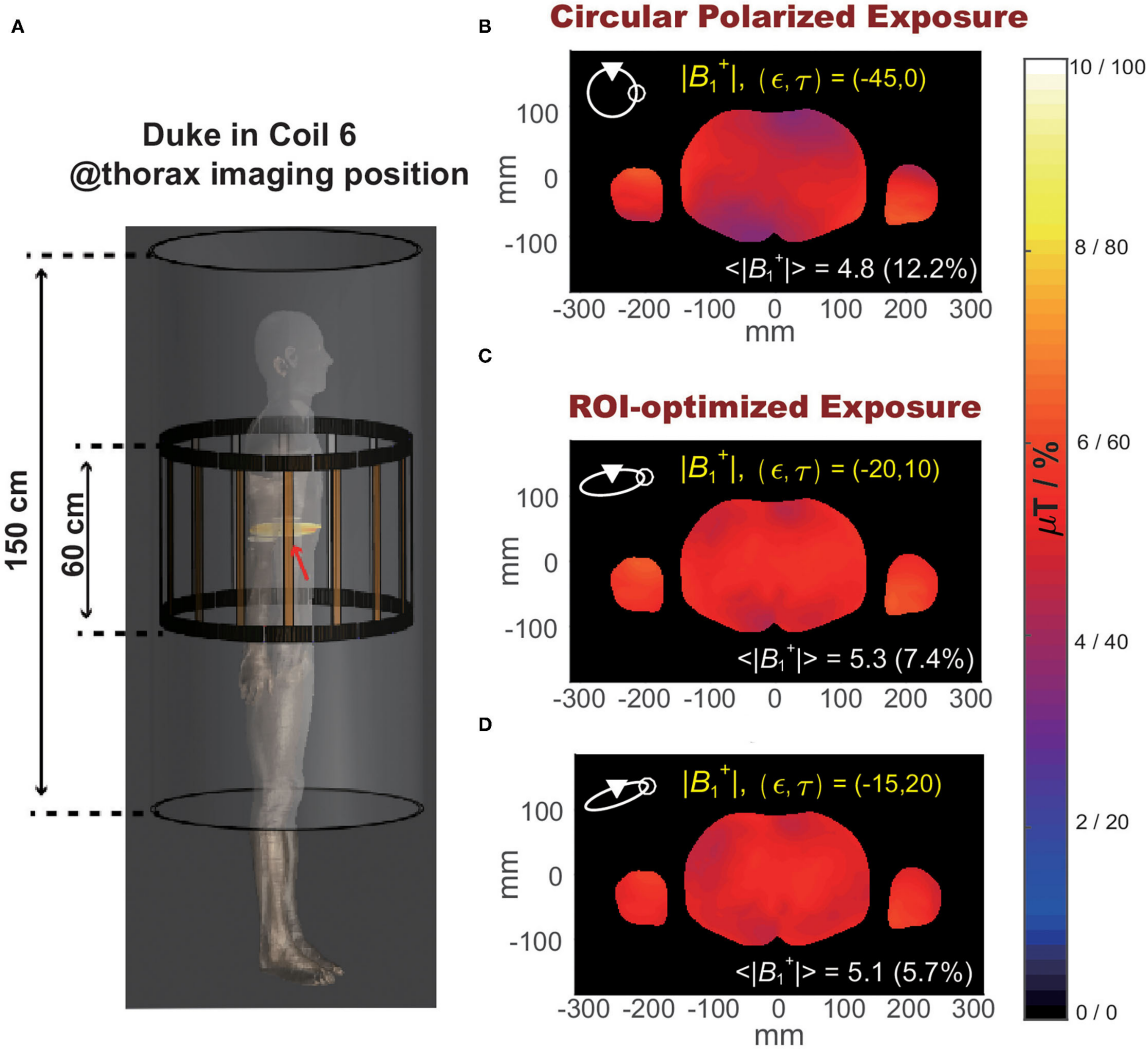
**(A)** Illustration of the selected clinical scenario: Duke in RF coil 6 at thorax imaging position. **(B)** Spatial distribution of $B_{1}^{+}$ magnitude at an iso-center slice of Duke under circular polarized *B*_1_ field. **(C)** Spatial distribution of $B_{1}^{+}$ magnitude at the iso-center slice of Duke under optimized exposure condition resulting in maximum $B_{1}^{+}$ magnitude. **(D)** Spatial distribution of $B_{1}^{+}$ magnitude over iso-center slice of Duke under optimized exposure condition resulting in minimum $B_{1,cov}^{+}$.

Figure 6 shows the slice view of the $\left\|B_{1}^{+}\right\|$ at an iso-center slice of an example clinical scenario: anatomical model Duke inside RF coil 6 at thorax imaging position (demonstrated in Figure 6A). Compared to the default circular polarized exposure (ϵ, τ)=(45^*o*^, 0) as shown in Figure 6B, the exposure allows maximum averaged $B_{1}^{+}$ field magnitude, shown in Figure 6C, improved averaged $B_{1}^{+}$ field magnitude $<\|B_{1,\left(\epsilon ,\tau \right)}^{+}\|>$ from 4.8 to 5.3 μT, while the exposure allows minimum $B_{1,cov}^{+}\left(\epsilon ,\tau \right)$, as shown in Figure 6D, decreased $B_{1,cov}^{+}\left(\epsilon ,\tau \right)$ from 12.2 to 5.7%.

**Figure 7:**
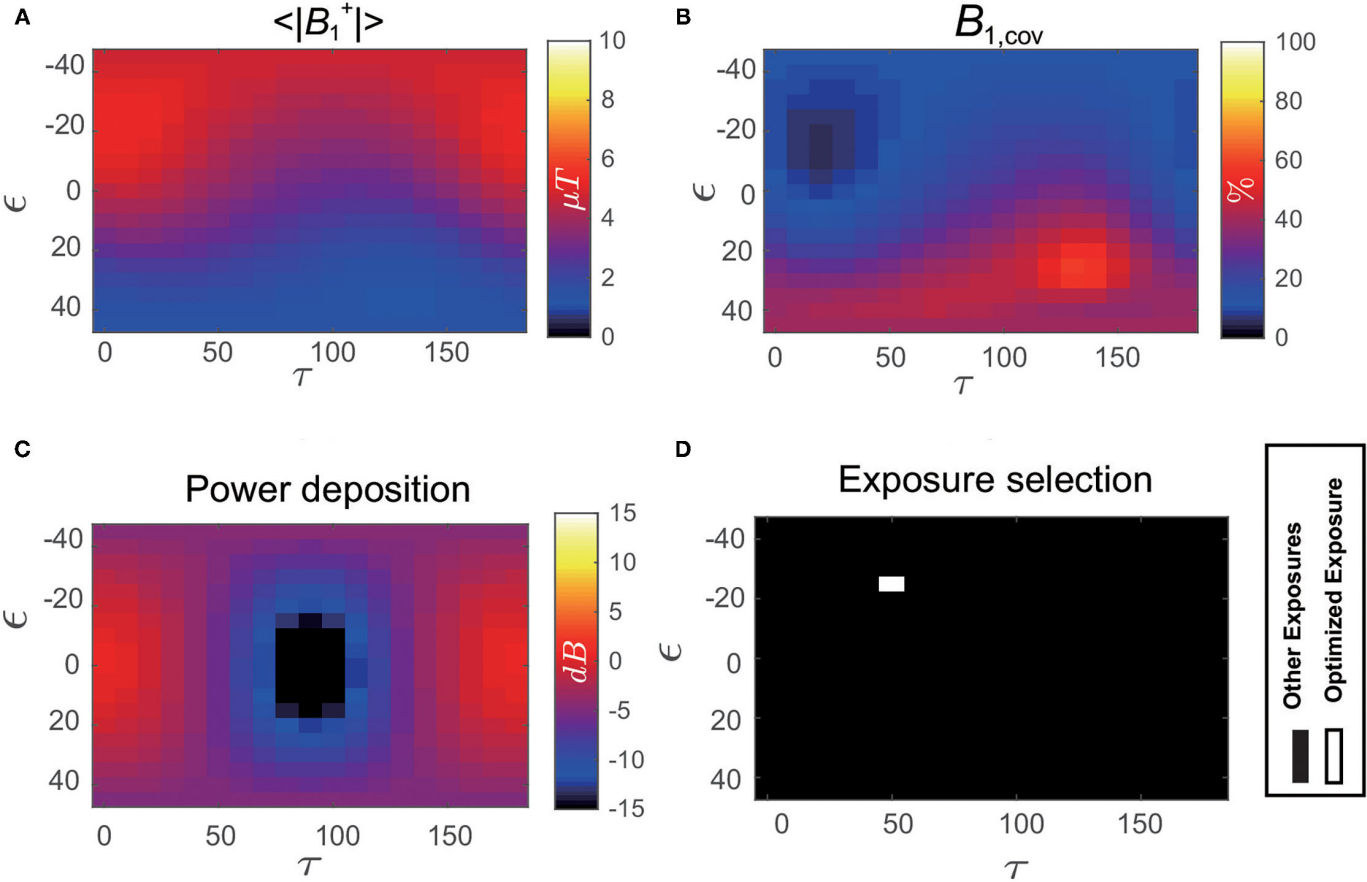
**(A)** Distribution of $B_{1}^{+}$ magnitude averaged over the iso-center slice of Duke as a function of *B*_1_ polarization (ϵ, τ). **(B)** Distribution of $B_{1}^{+}$ coefficient of variation $B_{1,cov}^{+}$ over the iso-center slice of Duke as a function of *B*_1_ polarization (ϵ, τ). **(C)** Distribution of *in vivo* power deposition (*P*_*dep*_) of the implant under test (IUT) implanted inside Duke as a function of *B*_1_ polarization (ϵ, τ). **(D)** Optimized exposure condition using the exposure optimization procedure performed on Duke under selected clinical scenario is shown as white.

Figure 7 shows the distribution of power deposition of the IUT as a function of the *B*_1_ polarization (*P*_*dep*_(ϵ, τ)) evaluated at the normal operating mode. The optimized exposure conditions that satisfy not only the image quality requirement (high $<\|B_{1}^{+}\|>$ and small $B_{1,cov}^{+}$) but also RF-induced heating limitation (low *P*_*dep*_) are shown as white in the bottom-right of Figure 7.

## Conclusion

In this work, we established an *in silico* exposure optimization trial that comprises a data library with a large permutation of different clinical scenarios to increase the evaluation completeness. To balance between the efficiency and completeness during the exposure optimization procedure, critical clinical factors are recognized and decoupled from the data library using principle component analysis. The proposed work-flow is applied to a generic 40-cm long active medical implant devices implanted in a 34-year-old male adult anatomical model as a pacemaker and exposed under 1.5T MRI RF magnetic field. The results show that the established workflow facilitates exploratory data analysis during exposure optimization, exposure conditions maximizing both imaging quality and patient safety under critical clinical scenarios can be identified.

## Data Availability Statement

The datasets presented in this article are not readily available because the dataset is licensed. Requests to access the datasets should be directed to https://itis.swiss/virtual-population/explib/overview/.

## Author Contributions

All authors listed have made a substantial, direct, and intellectual contribution to the work and approved it for publication.

## Funding

This work was supported by National Natural Science Foundation of China (Grant No. 6210010061).

## Conflict of Interest

The authors declare that the research was conducted in the absence of any commercial or financial relationships that could be construed as a potential conflict of interest.

## Publisher's Note

All claims expressed in this article are solely those of the authors and do not necessarily represent those of their affiliated organizations, or those of the publisher, the editors and the reviewers. Any product that may be evaluated in this article, or claim that may be made by its manufacturer, is not guaranteed or endorsed by the publisher.
